# Complete genome sequence of a lumpy skin disease virus isolate from a 2021 outbreak of disease in Bangladesh

**DOI:** 10.1128/mra.00667-24

**Published:** 2024-10-15

**Authors:** Mohammed A. Samad, Anowar Hossen, Md. Rezaul Karim, A. S. M. Ashab Uddin, Dipu Roy, Khairun Nahar Shithi, Mst Nazia Akter, Taposh Kumar Das, Paul W. Selleck, Dieter M. Bulach, Timothy R. Bowden

**Affiliations:** 1 Transboundary Animal Disease Research Center, Bangladesh Livestock Research Institute, Savar, Dhaka, Bangladesh; 2 Animal Health Research Division, Bangladesh Livestock Research Institute, Savar, Dhaka, Bangladesh; 3 Invent Technologies Ltd, Dhaka, Bangladesh; 4 CSIRO Australian Centre for Disease Preparedness, Geelong, Victoria, Australia; 5 The Asia-Pacific Centre for Animal Health, Melbourne Veterinary School, The University of Melbourne, Parkville, Australia; DOE Joint Genome Institute, Berkeley, California, USA

**Keywords:** complete genome sequence, lumpy skin disease virus, Bangladesh

## Abstract

Lumpy skin disease (LSD) is an economically devasting and vector-borne transboundary disease of cattle caused by lumpy skin disease virus (LSDV). Here, we report the complete genome sequence of an outbreak isolate of LSDV from Bangladesh. Bangladesh LSD-29 was detected in skin nodule samples of an LSD-infected bovine in 2021.

## ANNOUNCEMENT

Lumpy skin disease (LSD), a highly contagious, economically important transboundary viral disease of cattle that is transmitted mechanically by biting insect vectors, is caused by *Capripoxvirus lumpyskinpox* (ICTV: 2023 Release, MSL #39), and still referred to as lumpy skin disease virus (LSDV) within the genus *Capripoxvirus* and family Poxviridae (1). LSD was first detected in Bangladesh in 2019 (2). Here, we report the complete genome sequence of LSDV isolate Bangladesh LSD-29 (accession no. PP746705), which was isolated from skin nodule tissue samples in lamb testis cells during the LSD outbreak in cattle on 28 August 2021 at Kaliganj (23.411760°N, 89.129954°E), Jhenaidah district of Bangladesh. Following the first pass in lamb testis cell culture (3), DNA was extracted with the PureLink Viral DNA Mini Kit (Invitrogen, USA), according to the manufacturer’s instructions. The extracted DNA was measured with a Qubit dsDNA Quantification Assay Kit following the manufacturer’s instructions. The extracted DNA concentration was 12.1 ng/µL, of which 200 ng was used for sequencing. A sequencing library was prepared with Nextera DNA Flex for Illumina, USA. Libraries had an average fragment size of 400 bp. The Illumina NextSeq 2000 platform (2 × 150 bp chemistry) was used to sequence the libraries, generating 17.57 million reads. Library preparation and sequencing were performed at the Genome Sequence Laboratory, Bangladesh Livestock Research Institute, Savar, Dhaka. The quality of raw data was assessed using FastQC v0.11.3. Trimmomatic v0.39.2 (4) was used to remove adaptors and low-quality bases from reads using the following constraints: *Q* score >30; length >35 bp; 5′ clip = 15 (R1 and R2). Subsequently, LSDV reads were identified using Kraken2 (5) and extracted; this subset of 401,345 LSDV read-pairs (2.19% of the original read set) was assembled using SPAdes v3.13.2 with *k* values of 53, 65, 75, and 83; the final viral genome sequence was manually checked and confirmed (150,526 bp including a 2,295-bp inverted terminal repeat sequence and 160 ORFs); the average GC content for the genome was 25.9% (6). Using the annotation for LSDV isolate Kenya (accession MN072619) as a guide for product names, Prokka was used to produce a draft annotation of the genome sequence (7), which was manually curated prior to submission to NCBI. The Kenya isolate is a well-characterized LSDV isolate with a genome sequence highly related to isolate Bangladesh LSD-29. Differences between the isolates, including differences in encoded proteins, are listed in Table 1. Various types of genetic mutations, including single-nucleotide polymorphisms (snp), deletions (del), insertions (ins), multiple nucleotide polymorphisms (mnp), and complex mutations, were observed between LSDV isolates from Kenya and Bangladesh LSD-29. These mutations affect specific amino acid positions, leading to changes in protein structure and function.

**TABLE 1:** Differences between LSDV isolates Kenya and Bangladesh LSD-29

Accession^*a*^	POS^*b*^	Type^*c*^	REF^*d*^	ALT^*d*^	Effect^*e*^	Locus_tag	AA	Product
MN072619	6219	snp	T	A	Stop_lost; 163Tyr^*f*^	LSD-Kenya_010	162	Putative E3 ubiquitin ligase
MN072619	7940	del	AT	A	Intergenic			Intergenic
MN072619	12881	del	GT	G	Frameshift_variant; Lys229 frameshift	LSD-Kenya_019	569	Kelch-like protein
MN072619	18396	del	TA	T	Frameshift_variant; Leu105 frameshift	LSD-Kenya_026	299	Poxvirus_F11 superfamily protein
MN072619	23137	ins	A	AT	Frameshift_variant-start_lost; Met1 frameshift	LSD-Kenya_030	219	Pox_F16 superfamily protein
MN072619	75040	snp	C	A	Synonymous_variant; Ser63Ser	LSD-Kenya_083	786	NTPase
MN072619	87383	Complex	ATATGAGTG	TTATTAGTA	Missense_variant; HisIle536AsnAsn	LSD-Kenya_094	661	Hypothetical protein
MN072619	88739	snp	G	T	Missense_variant; Pro85His	LSD-Kenya_094	661	Hypothetical protein
MN072619	89709	mnp	AG	GA	Missense_variant; Arg23Glu	LSD-Kenya_096	170	DNA-dependent RNA polymerase subunit
MN072619	115875	del	TA	T	Intergenic			Intergenic
MN072619	132951	snp	T	A	Missense_variant; Asn203Lys	LSD-Kenya_140	240	E3 ubiquitin-protein ligase p28-like protein
MN072619	136065	ins	T	TTA	Frameshift_variant; Ser256frameshift	LSD-Kenya_144	547	Hypothetical protein
MN072619	137174	snp	G	A	Missense_variant; Glu59Lys	LSD-Kenya_145	634	Hypothetical protein

^*a*^ GenBank accession number of reference sequence.

^*b*^ POS, position of mutation in reference genome.

^*c*^ Type, mutation type; complex, complex difference.

^*d*^ REF, sequence in the Kenya isolate; ALT, sequence in the Bangladesh LSD-29 isolate.

^*e*^ Change in encoded protein; standard three letter amino acid code is used.

^*f*^ Indicates translation terminator.

## Data Availability

The complete genome sequence of LSDV isolate Bangladesh LSD-29 has been submitted to GenBank under accession number PP746705.1. The raw sequence reads have been submitted to the SRA database under accession number SRR29469192.
